# Should every adult patient in the hospital have an internist?

**DOI:** 10.1590/1516-3180.2013.1314719

**Published:** 2013-08-01

**Authors:** Mine Durusu Tanriover, Goksel Guven, Cagin Buldukoglu, Omer Diker, Burcin Halacli, Gonul Yildirim, Arzu Topeli

**Affiliations:** I MD. Associate Professor, Department of Internal Medicine, Hacettepe University Faculty of Medicine, Ankara, Turkey.; II MD. Resident, Department of Internal Medicine, Hacettepe University Faculty of Medicine, Ankara, Turkey.; III Nurse. Administrator, Adult Hospital Nursing Administration, Hacettepe University Faculty of Medicine, Ankara, Turkey.; IV MD. Professor, Department of Internal Medicine and Medical Intensive Care, Hacettepe University Faculty of Medicine, Ankara, Turkey.

Hospital medicine is a new venue for practicing internal medicine. It is the fastest growing specialty in the United States. While it has some similarities to “European” acute medicine, it differs particularly in that it provides continuous care throughout the hospitalization period and even beyond.^1^ Not only medical patients, but also surgical patients and patients in psychiatry and neurology wards are now being co-managed by hospitalists in the United States.^2^ Minor injuries might be managed by a nonsurgical hospitalist so as to improve the overall efficiency of the system.^3^

Patients admitted to non-internal medicine wards may even be in greater need of general medical care. We conducted a one-day cross-sectional survey to gather data on all the adult patients admitted to the wards (excluding intensive care and coronary care units and internal medicine wards) of a university hospital. There were 301 patients in the non-internal medicine, non-intensive care wards on a single day. The mean age of the patients was 50.5 ± 18.1 years (range: 16 to 92 years). More than 60% of the patients had been admitted for elective surgery (Table 1). Seventy-three percent of the patients (n = 220) presented at least one medical comorbidity and a median of two drugs (minimum 0; maximum 12) on the medication list (Table 1).

**Table 1.** Indications for admission and percentages of particular medical comorbidities amongst the study population (n = 301)

	% (n)
Reason for admission	
Elective surgery	62.1 (187)
Medical treatment	14.3 (43)
Investigation for differential diagnoses	12.0 (36)
Emergency surgery	4.7 (14)
Complications from surgery	4.0 (12)
Procedural treatment	2.0 (6)
Other	1.0 (3)
Medical comorbidity	
Hypertension	31.2 (94)
Cardiac disease	19.9 (60)
Diabetes	17.9 (54)
Malignancy	16.9 (51)
COPD/asthma	7.3 (22)
Renal dysfunction	6.0 (18)
Rheumatological disease	3.7 (11)
Transplantation	2.3 (7)
Infection	1.7 (5)
Chronic liver disease	1.3 (4)
Bedridden	0.7 (2)
Others*	23.6 (71)

Some patients presented more than one disease or condition. *Examples of other medical comorbidities included osteoporosis, osteoarthritis, gout and so on. No matter how many comorbidities in this group were present in a particular patient, they were taken to be “one” comorbidity. COPD = chronic obstructive pulmonary disease.

Recently, a Europe-wide study by Pearse et al. demonstrated that the mortality rate among patients who had undergone non-cardiac surgery was higher (4%) than expected.^4^ Perhaps more importantly, 73% of the patients who died had never been admitted to the intensive care unit. Accompanying diseases, such as cirrhosis, insulin diabetes and chronic obstructive pulmonary disease, were associated with mortality with odds ratios of up to 3.6. This reflects the vital importance of perioperative care for surgical patients, especially for those with medical comorbidities who would require effective, timely and high-quality general medical care. Hospitalists function as key members of patient-centered care during hospital admissions. Hospitalist co-management or consultation in nonmedical wards has been shown to shorten the length of time until surgery and the length of hospital stay, and to decrease the cost per stay.^5^

In conclusion, patients admitted to non-internal medicine wards present several medical comorbidities. Given the burden of chronic diseases in the aging population worldwide, the outcomes from implementing a hospitalist consultant or co-management system in non-medical wards should be investigated in prospective, large-scale studies.
